# Supplementary material for: Towards unified AI-driven fracture mechanics: the extended deep energy method (XDEM)
Source: Nat Commun. 2026 Aug 15;17:8492. doi: 10.1038/s41467-026-76748-1 (PMC13484471; doi:10.1038/s41467-026-76748-1)
Supplement: Supplementary file 3 [file 41467_2026_76748_MOESM3_ESM.zip › pic/Results/Crack_propagation/Straight/single_tension_centers.pdf]

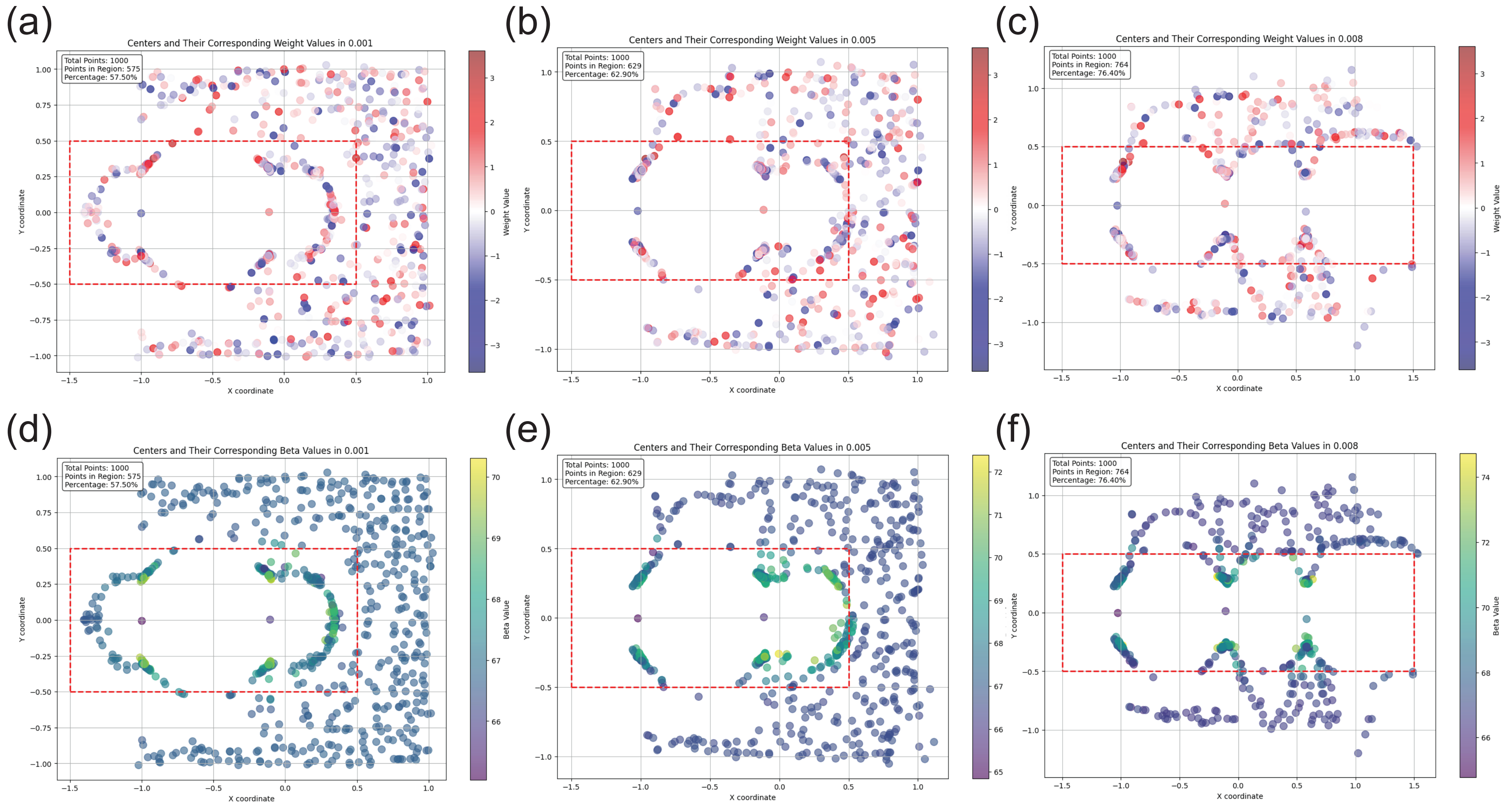
(a)
Centers and Their Corresponding Weight Values in 0.001
Total Points: 1000
Points in Region: 575
Percentage: 57.50%
Y coordinate
X coordinate
Weight Value
(b)
Centers and Their Corresponding Weight Values in 0.005
Total Points: 1000
Points in Region: 629
Percentage: 62.90%
Y coordinate
X coordinate
(c)
Centers and Their Corresponding Weight Values in 0.008
Total Points: 1000
Points in Region: 764
Percentage: 76.40%
Y coordinate
X coordinate
Weight Value
(d)
Centers and Their Corresponding Beta Values in 0.001
Total Points: 1000
Points in Region: 575
Percentage: 57.50%
Y coordinate
X coordinate
Beta Value
(e)
Centers and Their Corresponding Beta Values in 0.005
Total Points: 1000
Points in Region: 629
Percentage: 62.90%
Y coordinate
X coordinate
(f)
Centers and Their Corresponding Beta Values in 0.008
Total Points: 1000
Points in Region: 764
Percentage: 76.40%
Y coordinate
X coordinate
Beta Value
